# Quantitative Analysis of Flavonols and Their Glycosides and Gastrointestinal Transformation of Flavonol Glycosides in Yinxing Mihuan Oral Solution

**DOI:** 10.1155/jamc/1287338

**Published:** 2026-07-01

**Authors:** Yun Lyu, Wenqian Cheng, Kejing Niu, Shaoqing He, Lulu Ma, Jing Yang, Huijuan Yu

**Affiliations:** ^1^ State Key Laboratory of Chinese Medicine Modernization, Tianjin University of Traditional Chinese Medicine, No. 10 Poyanghu Road West Area Tuanbo New Town Jinghai District, Tianjin, 301617, China, tjutcm.edu.cn; ^2^ Xi’an Buchang TCM Cardiovascular and Cerebrovascular Hospital, No. 70 North Section of Labor Road Lianhu District, Xi’an, 710000, China; ^3^ Haihe Laboratory of Modern Chinese Medicine, No. 10 Poyanghu Road West Area Tuanbo New Town Jinghai District, Tianjin, 301617, China

**Keywords:** biotransformation, flavonol glycosides, gastrointestinal environment, network pharmacology, Yinxing Mihuan Oral Solution

## Abstract

Yinxing Mihuan Oral Solution (YMOS) is extensively utilized in the clinical treatment of cardiovascular and cerebrovascular diseases (CCVDs), with flavonols and their glycosides identified as the primary active ingredients. Currently, there are relatively few established quantitative methods available for analyzing flavonols and their glycosides, and the research regarding their transformation patterns under simulated gastrointestinal conditions is still limited. In this study, network pharmacology was employed to predict the active compounds and targets of YMOS associated with cardiovascular and cerebrovascular protection. A comprehensive network integrating compounds, targets, bioactivities, and CCVDs was meticulously constructed for 10 flavonol glycosides and their aglycones. It revealed that these compounds exert their blood circulation–promoting and blood stasis–eliminating effects by modulating 20 key targets, highlighting their therapeutic potential in CCVDs treatment. Furthermore, we quantitatively analyzed the 10 targeted flavonols and their glycosides in YMOS using ultra‐performance liquid chromatography (UPLC), demonstrating consistent flavonoid levels across all tested samples. This method was also employed to investigate biotransformation rules between flavonol glycosides and their aglycones in vitro. Notably, it was observed that flavonol glycosides undergo a deglycosylation reaction in the rat’s intestinal microbial culture medium, which was witnessed in a relatively stable manner under the simulated artificial gastrointestinal fluid. These findings provide important scientific insights into the mechanism of action and the quality control of YMOS.

## 1. Introduction

Cardiovascular and cerebrovascular diseases (CCVDs) have shown an accelerated increase in recent years due to changes in lifestyle and dietary habits, posing substantial challenges to public health and quality of life [1, 2]. As a result, there is an urgent need to find effective therapeutic approaches. Yinxing Mihuan Oral Solution (YMOS) is a formulation composed of ginkgo leaf extract and *Armillaria mellea* powder, which has emerged as a potential candidate for CCVDs treatment. The ginkgo leaf extract has the effect of promoting blood circulation, removing blood stasis, and unblocking collaterals [3], while *Armillaria mellea* can calm the nerves [4]. YMOS exhibits a variety of pharmacological properties, including reducing blood lipid levels and blood pressure and enhancing cardiocerebrovascular functions [5–8]. Specifically, it contributes to the maintenance of cholesterol homeostasis and the prevention of atherosclerosis by reducing total cholesterol (TC), triglycerides (TGs), and low‐density lipoprotein cholesterol (LDL‐C) levels and increasing high‐density lipoprotein cholesterol (HDL‐C) content [9, 10]. Previous studies have elucidated the protective effects of YMOS on the cardiocerebrovascular system. Xue et al. [11] employed network pharmacology to investigate the mechanism of action of YMOS in the protection of cardiocerebrovascular system and identified five active components that play a crucial role: quercetin, kaempferol, luteolin, Ginkgolide B, and Ginkgolide A. Guo et al. [12] further demonstrated that YMOS reduces the inflammatory response in the damaged myocardium, inhibits platelet activation, and provides myocardial protection in rats with myocardial ischemia. In addition, Song et al. [13] studied the protective effect of YMOS against cerebral ischemia‐reperfusion injury in rats, showing that it could diminish the extent of cerebral infarction, alleviate neurological damage, and safeguard brain neurons. Xu’s study, which employed a rat model of multiple cerebral infarction, demonstrated that YMOS can enhance neurological function [14]. Clinical studies have shown that YMOS significantly mitigates the frequency and duration of angina pectoris attacks in patients with coronary atherosclerotic heart disease [15]. Furthermore, Su et al. also reported that YMOS not only improves anxiety and depression but also enhances the quality of life for patients with coronary heart disease [16]. The principal active constituents of YMOS, which are derived from ginkgo leaf extracts, encompass flavonoids that are rich in glycosides of quercetin, kaempferol, and isorhamnetin [17–19]. Flavonoids are known for their diverse pharmacological activities, including enhancing vascular circulation, counteracting platelet‐activating factors, protecting the nervous system, exhibiting antioxidant properties, and displaying anticancer effects [20–23]. Li et al. [5] explored the therapeutic potential of ginkgetin in treating atherosclerosis in rat models, highlighting its role in modulating lipid metabolism and regulating MMP and NO/NOS systems. Moreover, Wang et al. [24] confirmed that ginkgo flavonoids markedly reduce the myocardial infarct area caused by ischemia, effectively treating acute myocardial ischemia. In summary, previous studies have confirmed the protective effects of YMOS on the cardiocerebrovascular system and the therapeutic prospects of its main flavonoid constituents.

YMOS is composed of flavonoids, terpenoid lactones, organic acids, polysaccharides, amino acids, and nucleosides [25, 26]. Yu et al. [27] developed an ultra‐high‐performance liquid chromatography tandem Q‐Exactive Orbitrap high‐resolution MS (UHPLC‐Q‐Orbitrap‐MS) method to identify 62 chemical constituents in YMOS, with nine principal compounds being quantitatively determined using UPLC coupled with triple quadrupole MS (UPLC‐QQQ‐MS). In our previous study, we identified 67 chemical compounds in YMOS using UHPLC/Q‐Orbitrap MS, including flavonoids, nucleosides, terpene lactones, and additives [28]. Among these compounds, five flavonol glycosides were quantified, and their stability exposed to temperature and pH was systematically studied by UPLC‐PDA. Although YMOS exhibits a promising pharmacological profile, the complexity of its chemical compositions and the paucity of fundamental research present hurdles for quality analysis. Moreover, the biotransformation of flavonol glycosides is commonly observed in traditional Chinese medicine in vivo, influencing the therapeutic efficacy of compounds such as naringin [29], hesperidin [30], and baicalin [31]. However, the biotransformation of flavonol glycosides from YMOS in simulated gastrointestinal conditions was not systematically investigated.

In the present study, we employed network pharmacology to investigate the putative active constituents and underlying action mechanisms of flavonols and their glycosides in YMOS for the management of CCVDs. Building upon this foundation, a multicomponent quantification method in YMOS was carried out using UPLC‐PDA. Moreover, an investigation was conducted into the biotransformation of flavonol glycosides derived from YMOS within artificial gastrointestinal fluid and a rat intestinal microbial culture medium. This comprehensive research not only deepens our understanding of the pharmacological basis of YMOS in CCVDs treatment but also provides a solid groundwork for the assessment of YMOS quality.

## 2. Materials and Methods

### 2.1. Reagents and Materials

LC‐grade methanol and acetonitrile were purchased from Sigma‐Aldrich Inc. (St. Louis, MO, USA). Formic acid was acquired from Shanghai Aladdin Biochemical Technology Co., Ltd. (Shanghai, China). Watsons’ water was purchased from Guangzhou Watsons Food & Beverage Co., Ltd. (Guangzhou, China). Hydrochloric acid was provided by Tianjin Jiangtian Chemical Technology Co., Ltd. (Tianjin, China). Sodium hydroxide and potassium dihydrogen phosphate were supplied by Tianjin Huihang Chemical Technology Co., Ltd. (Tianjin, China). ABS anaerobic culture medium was purchased from Qingdao High Tech Industrial Park Haibo Biotechnology Co., Ltd. (Qingdao, China). Pepsin and trypsin were obtained from Shanghai Yuanye Biotechnology Co., Ltd. (Shanghai, China). Ten batches of YMOS labeled as B1–B10 (10 mL per bottle) were gifted by Qionglai Tianyin Pharmaceutical Co., Ltd. (Sichuan, China). Reference standards including quercetin‐3‐*O*‐rutinoside (QGR), kaempferol‐3‐*O*‐rutinoside (KGR), isorhamnetin‐3‐*O*‐rutinoside (IGR), quercetin‐3‐*O*‐[2″‐(6″‐*p*‐coumaryl)‐*β*‐D‐glucosyl]‐*α*‐*L*‐rhamnoside (QRcG), quercetin, kaempferol, and isorhamnetin were offered by Shanghai Yuanye Bio‐Technology Co., Ltd. (Shanghai, China). Quercetin‐3‐*O*‐(2″‐*β*‐D‐glucosyl)‐*α*‐*L*‐rhamnoside (QRG), kaempferol‐3‐*O*‐(2″‐*β*‐D‐glucosyl)‐*α*‐*L*‐rhamnoside (KRG), and kaempferol‐3‐*O*‐[2″‐(6″‐*p*‐coumaryl)‐*β*‐D‐glucosyl]‐*α*‐*L*‐rhamnoside (KRcG) were offered by Chengdu Zhibiao Pure Biotechnology Co., Ltd. (Sichuan, China). The purities of all the above references were more than 98% by UPLC analysis.

### 2.2. Preparation of the Mixed Standard Solution and Tested Solution of YMOS

Ten reference standards were accurately weighed and respectively dissolved in methanol to prepare stock standard solutions. Afterward, the mixed stock solution was obtained by employing stock standard solutions of the tested compounds to reach the final concentrations at 128.16 μg/mL (QGR), 50.60 μg/mL (QRG), 70.70 μg/mL (KGR), 56.40 μg/mL (IGR), 167.28 μg/mL (QRcG), 50.30 μg/mL (KRG), 27.90 μg/mL (quercetin), 54.56 μg/mL (KRcG), 11.94 μg/mL (kaempferol), and 54.34 μg/mL (isorhamnetin). Subsequently, the mixed stock solution was serially diluted to obtain a series of working solutions with different concentrations, which were used to establish the standard curves. YMOS was diluted three times with 10% methanol and then centrifuged at 12,000 rpm for 10 min before UPLC analysis. All solutions were stored at 4°C for further analysis.

### 2.3. UPLC‐PDA Conditions and Methodological Validation of the Quantitative Analysis

An ACQUITY UPLC I class system (Waters Corporation, Milford, MA, USA) was used to perform the quantification of the 10 flavonoids in YMOS. Chromatographic separation was carried out on an ACQUITY UPLC HSS T3 column (2.1 × 100 mm, 1.7 μm, Waters, Milford, MA, USA) by employing 0.2% formic acid aqueous solution (*A*) and methanol (B) as the mobile phase in a gradient elution (50°C) at a flow rate of 0.3 mL/min as follows [28]: 0–3 min, 6%–14% B; 3–6 min, 14%–23% B; 6–8.3 min, 23%–30.5% B; 8.3–10.3 min, 30.5%–33.5% B; 10.3–12.5 min, 33.5%–41.5% B; and 12.5–19 min, 41.5%–74% B. The detection wavelength was set at 254 nm. The injection volume of each sample was 2 μL.

For testing the feasibility of the analytical method for quantitative analysis of the tested compounds, linearity, limit of detection (LOD), limit of quantification (LOQ), precision (intra‐ and interday), repeatability, stability, and recovery test were systematically validated. The calibration curves were constructed according to the peak areas (*y*) and the corresponding concentrations (*x*) of the tested compounds. The concentrations of each analyte at signal‐to‐noise (S/N) ratios of 3 and 10 were defined as the LOD and LOQ, respectively. The intra‐ and interday precisions were assessed by performing six tests on the same sample solution within a single day and on three parallel sample solutions over three consecutive days, respectively. Repeatability was evaluated with six independently prepared sample solutions. The stability was evaluated by analyzing the same sample solution at 0, 2, 4, 6, 8, 10, and 12 h within one day. The recovery was investigated by analyzing six sample solutions processed by adding the reference solution into precisely measured 0.5 mL YMOS.

### 2.4. Preparation of the Artificial Gastrointestinal Fluid and the Rat’s Intestinal Microbiota Culture Medium

We prepare artificial gastric and intestinal fluids according to the methods provided in the Chinese Pharmacopoeia (2020) [3]. The preparation of simulated artificial gastric juice was performed as follows: 16.4 mL (234 mL of hydrochloric acid diluted to 1000 mL with water) of diluted hydrochloric acid was taken and diluted with water to a final volume of 800 mL. Subsequently, 10 g of pepsin was accurately weighed and added to the diluted hydrochloric acid solution. The mixture was shaken thoroughly, and its pH was adjusted to 1.3 using 0.1 mol/L hydrochloric acid solution. Finally, the solution was diluted with water to 1000 mL to obtain the simulated artificial gastric juice.

For the preparation of simulated artificial intestinal juice, 6.8 g of potassium dihydrogen phosphate was weighed and dissolved completely in 500 mL of water. The pH of this solution was then adjusted to 6.8 using a 0.1 mol/L sodium hydroxide solution. Separately, 10 g of trypsin was accurately weighed and dissolved in an appropriate amount of water. Afterward, the two solutions were combined and diluted with water to 1000 mL, thereby obtaining the simulated artificial intestinal juice.

The fresh feces from healthy rats were mixed with physiological saline in a ratio of 1:5 (*w*/*v*, g/mL). After centrifugation at 4000 rpm, the supernatant was diluted with the quintuple volume of anaerobic culture medium and mixed well to obtain the rat’s intestinal microbial culture medium [32].

### 2.5. Preparation of Samples for Biotransformation in Simulated Gastrointestinal Environment

Accurately, YMOS (6 mL) was respectively added to artificial gastric juice (24 mL) and intestinal juice (24 mL), which were incubated at 37°C. At the different incubation time points (0, 2, 4, 6, 8, 10, and 12 h), 300 μL of the sample solution was collected and diluted with 700 μL of acetonitrile, respectively. The mixture was centrifuged at 14,000 rpm for 10 min to obtain the supernatant for analysis.

YMOS (6 mL) was added to the rat’s intestinal microbial culture medium (36 mL) and incubated at 37°C for 24 h. Sample solution (200 μL) was collected at the different incubation time points (0, 3, 6, 9, 12, 18, and 24 h), and mixed with an equal volume of acetonitrile. After centrifugation (4000 rpm, 10 min), the supernatant was employed for further analysis. The control group solutions were prepared using physiological saline according to the same method.

### 2.6. Network Pharmacology Analysis

The structures of the 10 focused compounds from YMOS were downloaded from the PubChem database (https://pubchem.ncbi.nlm.nih.gov/) and uploaded into the SwissTargetPrediction database (http://www.swisstargetprediction.ch/) [33] to predict potential targets. The screening condition was limited to “*Homo sapiens,*” and potential active targets (probability^∗^ > 0) were collected after the removal of duplicates. Coronary atherosclerotic heart disease, angina pectoris, and ischemic cerebrovascular disease, which are the representative CCVDs for YMOS, along with “promoting blood circulation to remove blood stasis,” the main bioactivity of YMOS, was respectively used to search for relevant targets in the GeneCards database (https://www.genecards.org/). The targets with a relevance score ≥ 1 were selected as primary targets associated with the diseases and bioactivity, respectively. The common targets were identified by taking the intersection of the targets related to CCVDs and those related to the compounds. These common targets were further intersected with the targets associated with the bioactivity of YMOS to obtain the shared targets among the compounds, CCVDs, and the bioactivity. A compounds‐targets‐bioactivity‐CCVDs network was constructed using Cytoscape (Version 3.7.1). The identified shared targets were submitted to the STRING 11.5 database (https://www.string-db.org/) to retrieve interaction data. Subsequently, these interaction data were visualized in a protein–protein interaction (PPI) network using Cytoscape software. The species “*Homo sapiens*” was selected, and the minimum required interaction score was set to 0.4 to improve the accuracy of the results. In addition, these targets were imported into the DAVID 2021 database (https://david.ncifcrf.gov/) for Gene Ontology (GO) enrichment and Kyoto Encyclopedia of Genes and Genomes (KEGG) pathway enrichment analysis with *p* < 0.05 as the screening condition, respectively.

### 2.7. Data analysis

The line chart and heatmap were created using OriginPro 2024 (Learning Edition) software (OriginLab Ltd., Northampton, MA, USA) and Adobe Illustrator 2020 (Adobe, San Jose, CA, USA).

## 3. Results

### 3.1. The Focused Compounds‐Targets‐Bioactivity‐CCVDs Network Predicted by Network Pharmacology

In our previous research [28], we characterized the chemical components of YMOS, among which we focused on the main 10 flavonoids composed of quercetin, kaempferol, isorhamnetin, and their glycosides (QGR, QRG, KGR, IGR, QRcG, KRG, and KRcG) in this study. In order to elucidate their mechanisms of action in the treatment of CCVDs, we conducted a network pharmacology analysis. 120 potential targets of the 10 flavonoids were retrieved from SwissTargetPrediction after removing duplicate targets. Using GeneCards, we obtained 3087, 2115, and 2241 targets for coronary atherosclerotic heart disease, angina pectoris, and ischemic cerebrovascular disease, respectively, as well as 443 targets associated with promoting blood circulation to remove blood stasis. By employing Venny (2.1.0), we acquired 34 common targets shared by the compounds and the focused diseases and further concentrated 20 targets of compounds‐CCVDs‐bioactivity.

**FIGURE 1:**
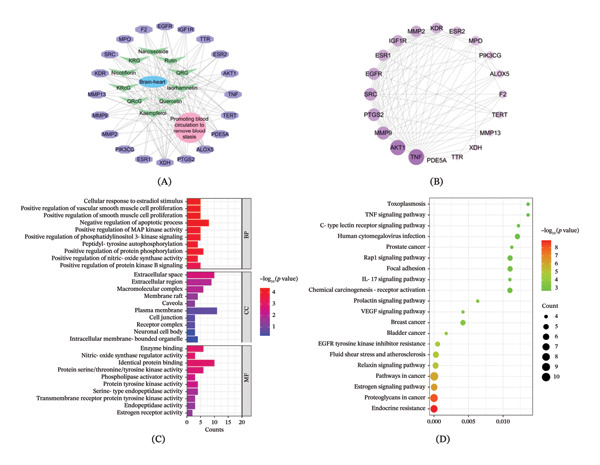
(A) Compounds‐targets‐bioactivity‐CCVDs network. (B) PPI network of target proteins. (C) Histogram of GO enrichment analysis. (D) Bubble chart of KEGG enrichment analysis.

Cytoscape 3.7.1 software was employed to construct the compounds‐targets‐bioactivity‐CCVDs network to elucidate the complex interactions among compounds, bioactivity, and CCVDs through the related targets (Figure 1A). As shown in Figure 1B, we identified the key targets, such as TNF, AKT1, and MMP9, which play significant roles in the PPI network based on degree values. We conducted GO enrichment analysis to analyze the biological information of the targets (Figure 1C), involving biological processes (BPs), cellular components (CCs), and molecular functions (MFs). As shown in Figure 1C, we discovered that the focused compounds in YMOS may have certain effects in regulating cytokine‐related BPs, such as the “negative regulation of apoptotic process.” Previous research has indicated that regulating apoptosis is a promising option for treating cardiovascular diseases and conditions such as myocardial infarction, ischemia/reperfusion injury, and heart failure [34], providing evidence for the treatment of CCVDs with YMOS. Furthermore, KEGG enrichment analysis was also performed (Figure 1D) to cluster the main pathways, indicating that the core pharmacological mechanisms of flavonoids from YMOS are associated with endocrine resistance, proteoglycans in cancer, estrogen signaling pathway [35–37], and pathways in cancer [38, 39]. These findings suggest that YMOS may exert its therapeutic effects on CCVDs through multiple pathways and targets [40].

### 3.2. Methodological Validation and Quantification of Ten Flavonoids in YMOS

**FIGURE 2:**
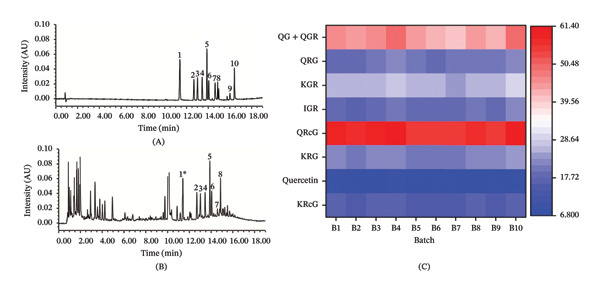
UPLC‐PDA chromatograms of (A) the mixed standard solution and (B) YMOS sample solution. (C) The heatmap of the contents for multicompounds in 10 batches of YMOS (1∗. QG + QGR; 2. QRG; 3. KGR; 4. IGR; 5. QRcG; 6. KRG; 7. quercetin; 8. KRcG; 9. kaempferol; 10. isorhamnetin).

The 10 flavonoids can be satisfactorily separated in 19 min by the established method, whose typical chromatograms are shown in Figure 2A,B. Due to the almost identical chromatographic retention behavior of isoquercetin (QG) and QGR, their chromatographic peaks highly overlapped [28], which originates from the same aglycone (quercetin). In order to determine the total content of QG and QGR, we chose QGR as the reference substance in this part. The validation results of the simultaneous quantification method for 10 flavonoids are shown in Table 1. As a result, the 10 compounds had excellent linear relationships within the tested concentration range. The LOD and LOQ values were in the range of 0.05–0.52 μg/mL and 0.07–0.65 μg/mL, respectively. The RSDs of the intra‐ and interday precisions, stability, and repeatability were proved to be below 4.00%. In addition, the average recoveries of the tested compounds were 95.69%–113.81% with all RSDs below 2.11%. In general, the established UPLC‐PDA method is reasonable and feasible for multicomponent quantitation analysis of YMOS.

**TABLE 1:** Summary results of linear regression, LODs, precision, repeatability, stability, and recovery for the 10 tested compounds in YMOS.

Compounds	Linear regression			LODs (μg/mL)	Precision (RSD, %)		Repeatability (*n* = 6, RSD, %)	Stability (*n* = 7, RSD, %)	Recovery (*n* = 6, mean ±SD, %)
	Regression equation	*r* ^2^	Linear range (μg/mL)		Intraday (*n* = 6)	Interday (*n* = 6)			
QGR	*y* = 13,679.00 *x* + 4897.81	0.9999	4.00–128.16	0.40	1.07	0.28	0.39	1.14	106.08 ± 0.95
QRG	*y* = 13,648.77 *x* + 9924.50	0.9998	1.58–50.60	0.16	1.09	0.67	1.08	1.37	95.69 ± 1.25
KGR	*y* = 10,957.12 *x* +2106.99	0.9999	2.21–70.70	0.22	0.63	0.59	0.55	0.87	98.38 ± 1.59
IGR	*y* = 13,593.65 *x* + 5772.79	0.9999	1.76–56.40	0.18	0.33	0.49	1.07	0.66	101.31 ± 1.43
QRcG	*y* = 9980.67 *x* +16,943.22	0.9999	5.23–167.30	0.52	0.38	1.77	0.47	1.10	96.41 ± 1.47
KRG	*y* = 11,049.96 *x* + 4729.52	0.9999	1.57–50.30	0.16	0.27	1.70	0.72	1.88	99.98 ± 0.75
Quercetin	*y* = 13,644.06*x* −11,311.58	0.9996	0.87–27.90	0.11	0.67	2.97	0.61	4.00	113.81 ± 1.70
KRcG	*y* = 18,872.46 *x* + 3555.48	0.9999	1.71–54.56	0.21	0.69	2.76	0.53	2.65	98.70 ± 2.08
Kaempferol	*y* = 20,363.87 *x* − 1318.09	0.9998	0.37–11.94	0.05	—	—	—	—	105.59 ± 0.42
Isorhamnetin	*y* = 19,554.87 *x* − 6766.06	0.9999	1.70–54.34	0.21	—	—	—	—	97.11 ± 0.68

*Note:* “–”, N.D.

Subsequently, we applied the established method for quantitative detection across multiple batches of YMOS. The contents range of these flavonoids in 10 batches of YMOS was 6.84–61.27 μg/mL. Among them, the content of QRcG (55.07–61.27 μg/mL) was found to be the highest, while the content of quercetin (6.84–8.87 μg/mL) was the lowest. Neither kaempferol nor isorhamnetin was detected, primarily due to the presence in the form of their glycosides in YMOS. To visually represent the variations in multicomponent contents in different batch samples, the heatmap was utilized in this study (Figure 2C). The redder the color is, the higher the content is, whereas the bluer the color is, the lower the content is. The results demonstrate that the concentrations of individual bioactive compounds in YMOS exhibit remarkable consistency across multiple production batches, suggesting both stable raw material quality and stringent process control throughout manufacturing.

### 3.3. Biotransformation of Flavonol Glycosides in the Simulated Gastrointestinal Environment

Oral administration is one of the most important forms in traditional Chinese medicine. After oral administration, glycosides in traditional Chinese medicine have been linked to contact with gastric juice, intestinal juice, and gut microbiota in the gastrointestinal tract. This interaction leads to the degradation and metabolic transformation of the target compounds before they are absorbed into the bloodstream, ultimately influencing their therapeutic efficacy [32, 41]. Herein, we investigated the biological transformation of seven flavonol glycosides derived from YMOS in simulated gastrointestinal environments, specifically artificial gastric juice, artificial intestinal juice, and a culture medium of rat intestinal microbiota. This study aims to elucidate the biotransformation mechanism.

As displayed in Figures 3A and B, the seven flavonol glycosides (QGR, KGR, IGR, QRcG, QRG, KRG, and KRcG) derived from YMOS remained stable over time in artificial gastric juice. When incubated in alkaline artificial intestinal juice, QRcG and KRcG were observed to gradually degrade, with degradation rates of 30.73% and 22.85%, respectively. QRG and KRG showed an increasing trend, while QGR, KGR, and IGR showed no significant change (Figures 3C and D). Collectively, the tested flavonol glycosides were less affected by the gastrointestinal environment, except for QRcG and KRcG. It suggested that lactone bonds of QRcG and KRcG are subject to alkaline conditions, leading to the cleavage (Figure 3E). The phenolic hydroxyl groups impart slight acidity to the compounds, enhancing their stability under acidic conditions [42, 43].

FIGURE 3(A) Chromatograms of the biotransformed sample solutions in artificial gastric juice. (B) Time‐concentration curves of the focused compounds in artificial gastric juice (*n* = 3). (C) Chromatograms of the biotransformed sample solutions in artificial intestinal juice. (D) Time‐concentration curves of the focused compounds in artificial intestinal juice (*n* = 3). (E) Biotransformation rules of QRcG and KRcG (1∗. QG + QGR; 2. QRG; 3. KGR; 4. IGR; 5. QRcG; 6. KRG; 8. KRcG).

**Figure:**
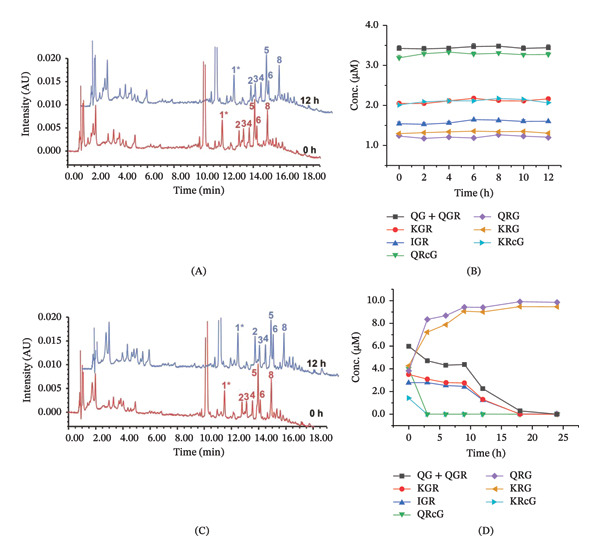

**Figure:**
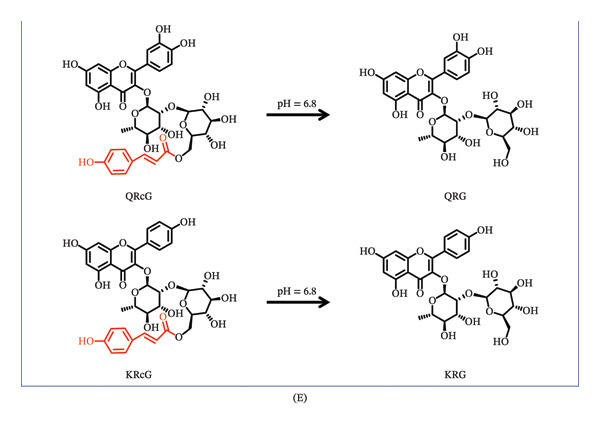

**FIGURE 4:**
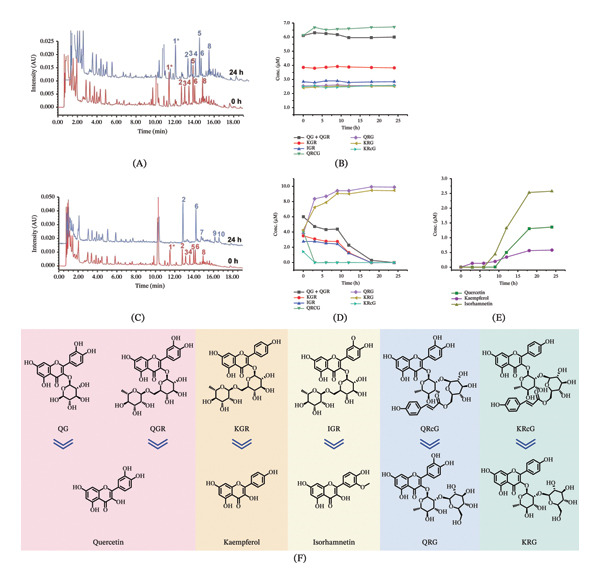
(A) Chromatograms of the biotransformed sample solutions in the control group. (B) Time‐concentration curves of the focused compounds in the control group (*n* = 3). (C) Chromatograms of the biotransformed sample solutions in the rat’s intestinal microbiota culture medium. (D, E) Time‐concentration curves of the focused compounds in the rat’s intestinal microbiota culture medium (*n* = 3). (F) Biotransformation rules in rat’s intestinal microbiota culture medium (1∗. QG + QGR; 2. QRG; 3. KGR; 4. IGR; 5. QRcG; 6. KRG; 7. quercetin; 8. KRcG; 9. kaempferol; 10. isorhamnetin).

As shown in Figures 4A and C, significant biotransformation of the seven flavonol glycosides derived from YMOS occurred after 24 h incubation in rat intestinal microbiota culture medium when compared to the control group. It is noteworthy that all compounds in the control group maintained stability throughout the incubation period (Figure 4B). In contrast, within the rat intestinal microbiota culture medium, QRcG, KRcG, QGR, KGR, and IGR were observed to undergo significant degradation. Conversely, the level of KRG and QRG increased (Figure 4D). Quercetin, kaempferol, and isorhamnetin were detected at 12, 3, and 9 h during incubation, respectively (Figure 4E). Through the analysis of the compound structures, QRG and KRG were formed by hydrolyzing coumaryl groups from QRcG and KRcG, respectively. Quercetin, kaempferol, and isorhamnetin were produced by hydrolyzing rutinose from QGR, KGR, and IGR, respectively. Possibly, QRG and KRG could undergo hydrolyzation to produce quercetin and kaempferol. Generally, flavonol glycosides undergo deglycosylation and hydrolysis reactions in the rat intestinal microbiota culture medium, leading to the formation of their aglycones. In summary, the investigated flavonol glycosides demonstrate considerable stability in gastrointestinal fluid, whereas their biotransformation is predominantly governed by intestinal microbiota, providing critical insights into their in vivo pharmacological mechanisms.

## 4. Discussion

At present, network pharmacology has been increasingly utilized to study traditional Chinese medicine formulas, elucidate the interactions between drugs and diseases, and predict the active ingredients and their mechanisms of action [44]. In the clinic, YMOS has emerged as a promising therapeutic option for the treatment of CCVDs [45, 46]. As shown in Figure 1A, the larger green arrow represents the higher degree values, indicating greater importance of the components. Kaempferol, quercetin, and isorhamnetin were suggested as the main active flavonoids contributing to the therapeutic effects in YMOS. PPI analysis presented that TNF, AKT1, and MMP9 are the key targets for the treatment of CCVDs by YMOS. As an important active form of TNF, TNF‐*α*, a peptide abundantly present in the body, is considered as a major participant in the pathophysiology of myocardial cells [47]. It has high expression in patients with myocarditis. Research studies have indicated that quercetin can mitigate TNF‐*α*‐induced apoptosis and inflammation in H9c2 cells by inhibiting the STAT1 and MAPK pathways, thereby protecting myocardial cells [48]. In addition, kaempferol can prevent the production of TNF‐*α* by inhibiting the expression of the TLR4/NF‐*κ*B pathway, thus playing a neuroprotective role against cerebral ischemic reperfusion injury [49]. AKT is a protein kinase involved in critical processes such as cell survival and apoptosis, playing a significant role in cardiovascular diseases such as atherosclerosis [50] and myocarditis [51]. Kaempferol exerts a protective effect on atherosclerosis by inducing upregulation of GPER to regulate the PI3K/AKT/Nrf2 pathway [52]. Similarly, isorhamnetin can also activate the PI3K/AKT pathway to prevent the formation and development of atherosclerotic plaque in arteries [53]. MMP9 is a biomarker for unstable atherosclerotic plaques in arteries [54]. Quercetin could reduce MMP9 levels to treat atherosclerosis [55]. Generally, flavonoids in YMOS perform a synergistic effect on the treatment of CCVDs through multiple components, targets, and pathways.

Flavonoid glycosides can be hydrolyzed by glycosidases secreted by the gut microbiota, resulting in the production of secondary glycosides and aglycones, which are subsequently converted into glucuronidation and methylation products by Phase II metabolic enzymes [56]. The metabolic pathways of flavonol glycosides with different structures exhibit notable differences. For example, flavonoid *O*‐glycosides can undergo deglycosylation through *β*‐glucosidase secreted by gut microbiota, whereas flavonoid C‐glycosides can only be hydrolyzed by specific enzymes produced by the gut microbiota [57]. In this study, we investigated the conversion of flavonol glycosides from YMOS in a simulated gastrointestinal environment. The results revealed that flavonol *O*‐glycosides could be converted into their corresponding aglycones in the rat’s intestinal microbiota culture medium, which may be attributed to the cleavage of glycosidic bonds by *β*‐glucosidase from the gut microbiota [58–60]. These aglycones have also been demonstrated to have important biological activities in the treatment of CCVDs [48, 49, 52, 53, 55]. Based on it, the quality control indicators were focused on taking the activity of the exposed ingredients, the prices, and the availability into account. According to our accomplished study, we propose to further simplify the quantitative markers, conducing to the expanding application in the pharmaceutical factory. Collectively, the three main aglycones (quercetin, kaempferol, and isorhamnetin) can be adopted as the quantitative markers through acid hydrolysis under heating reflux conditions [3, 60], which is simpler, more sensitive, and feasible. Importantly, novel analytical methods must be undertaken to accomplish quantification of the effective compounds in YMOS, except for flavonoids.

## 5. Conclusion

This study established an integrated compounds‐targets‐bioactivity network to elucidate the CCVDs therapeutic mechanisms of YMOS via network pharmacology. We develop a robust UPLC‐PDA method quantifying simultaneously 10 flavonoids, demonstrating exceptional batch‐to‐batch consistency of YMOS. Crucially, the biotransformation of flavonol glycosides in the gastrointestinal environment was systematically investigated, offering new insights into the action mechanism in vivo and the quality control paradigm of YMOS.

## Author Contributions

Yun Lyu, Wenqian Cheng, Jing Yang, and Huijuan Yu conceived the experiment and designed the study. Wenqian Cheng and Yun Lyu performed all the other experiments. Kejing Niu participated in the methodology research. Shaoqing He and Lulu Ma analyzed the data. Wenqian Cheng, Jing Yang, and Huijuan Yu contributed to the writing of this article.

## Funding

This research was supported by the Science and Technology Program of Tianjin (Grant no. 25ZYCGCG00390), the Science and Technology Project of Haihe Laboratory of Modern Chinese Medicine (Grant no. 25HHZYSS00005 and 25HHZYSS00006), the Special Project for Technological Innovation in New Productive Forces of Modern Chinese Medicines (Grant no. 24ZXZKSY00010), and the Science and Technology Project of Haihe Laboratory of Modern Chinese Medicine (Grant no. 25HHZYSS00024).

## Disclosure

All authors read and approved the final manuscript.

## Conflicts of Interest

The authors declare no conflicts of interest.

## Data Availability

The data used to support the findings of this study are included within the article.
